# Menstruation-related symptoms are associated with physical activity and midpoint of sleep: a pilot study

**DOI:** 10.3389/fgwh.2023.1260645

**Published:** 2023-12-20

**Authors:** Hazuki Masuda, Shima Okada

**Affiliations:** ^1^ Biophysical Engineering Lab, Faculty of Science and Engineering, Ritsumeikan University, Shiga, Japan; ^2^ Biophysical Engineering Lab, Department of Robotics, Faculty of Science and Engineering, Ritsumeikan University, Shiga, Japan

**Keywords:** menstruation-related symptoms, physical activity, sleep, menstrual cycle, menstrual cycle phase, generalized linear mixed model, wearable device

## Abstract

**Introduction:**

Menstruation-related symptoms (MRSs) significantly impact women's health and contribute to economic burdens worldwide. Current interventions, primarily pharmacological ones, have limitations and side effects that underscore the need for alternative management strategies. This study explores the association between MRSs and lifestyle factors, specifically physical activity and sleep timing across menstrual cycle phases, to inform non-pharmacological intervention development.

**Methods:**

Fourteen female students from Ritsumeikan University, Japan, with regular menstrual cycles (25–38 days), not on hormonal treatment or engaged in shift work, participated in this observational study. Using a Fitbit Inspire 2, total daily energy expenditure (TDEE) and sleep timing were monitored over a complete cycle. Menstrual cycle phases were defined based on ovulation day, predicted using home luteinizing hormone tests. Participants completed daily electronic questionnaires rating MRSs using a modified menstrual distress questionnaire. Data were analyzed using a generalized linear mixed model with a gamma distribution and logarithmic link function, examining the relationship of TDEE and the midpoint of sleep time (MS time) with MRS severity.

**Results and discussion:**

The following observations were noted: first, MRS severity, except for behavioral change symptoms, significantly increased during the menstrual and luteal phases compared to the follicular phase. Second, delayed MS time was associated with reduced pain, concentration symptoms, water retention, and negative affect during the menstrual phase and reduced negative affect during the luteal phase. Finally, an increase in TDEE was associated with reduced concentration symptoms, autonomic reaction symptoms, and negative affect during the menstrual and luteal phases and reduced water retention only during the luteal phase. This study provides insights into the relationship between MRSs and TDEE/MS time, suggesting potential non-therapeutic approaches for symptom management, though further research is needed to substantiate these findings for practical applications.

## 1. Introduction

Hormonal fluctuations associated with the menstrual cycle affect women's health. Menstruation-related symptoms (MRSs) include heavy menstrual bleeding, dysmenorrhea with lower abdominal pain associated with menstrual bleeding, and premenstrual syndrome, in which physical and mental discomfort persists for 3–10 days before menstrual bleeding. Sleep quality subjectively deteriorates during the menstrual and luteal phases in women with menstrual pain and premenstrual syndrome (1, 2). Objectively measured sleep quality has been found to be impaired in women with premenstrual syndrome, particularly premenstrual dysphoric disorder (PMDD) (3). MRSs are significant, leading to reduced academic performance and an increased economic burden due to decreased labor productivity (4, 5). Therefore, addressing MRSs transcends individual health, bearing significant implications for societal productivity and economic burden.

A survey conducted by Japan's Ministry of Health, Labor and Welfare of 3,000 women revealed that enduring symptoms and taking over-the-counter medications are common ways of coping with MRSs, and that the rate of visits to a medical institution is only around 10% (6). Painkillers and hormonal therapy are effective in alleviating MRSs, while nonsteroidal anti-inflammatory drugs relieve primary dysmenorrhea (7). Meanwhile, some individuals express concerns regarding the drug regimen and its associated side effects. Therefore, there is a need to develop non-drug methods to alleviate MRSs.

Recent studies have suggested exercise and light therapy approaches for alleviating MRSs. Exercise effectively improves physical symptoms, such as pain, constipation, and breast sensitivity, and mental symptoms, such as anxiety and anger (8, 9). Aerobic exercise has been reported to relieve dysmenorrhea (10). This effect is thought to be due to the fact that exercise stimulates estradiol secretion in the body and serotonin secretion in the brain (11, 12). Since an acrophase of circadian rhythms for patients with PMDD are advanced, light therapy was effective in significantly reducing their depressive symptoms (13). On the other hand, inappropriate sleep timing (social jet lag and shift work) may exacerbate these circadian rhythm disturbances and worsen MRSs (14).

While there has been significant research into the relationship between MRSs and physical activity/sleep timing, several methodological limitations persist in existing literature. Notably, many studies have relied on retrospective self-reports of symptoms, which are susceptible to recall bias and may not accurately capture the nuances of daily menstrual experiences. Furthermore, few have provided an integrated approach that considers physical activity, sleep timing, and MRSs within the context of the menstrual cycle phase (MCP) using daily data.

Our objective was to investigate the relationship between the severity of MRSs and total daily energy expenditure (TDEE) as physical activity and the midpoint of sleep time (MS time) according to the MCP. Our hypothesis was that increased TDEE, and delayed MS time during the menstrual and luteal phases are related to reduced MRSs. Establishing this relationship can pave the way for personalized exercise and sleep timing recommendations tailored to individual MCPs, thereby alleviating MRSs.

## 2. Methods

### 2.1. Participants

This study included 14 female undergraduate and graduate students at Ritsumeikan University, Japan. Participants were eligible for the study if they self-reported that they were not currently receiving treatment for sleeping disorders, had a menstrual cycle of 25–38 days (definition of a normal menstrual cycle by the Japanese Society of Obstetrics and Gynecology), were not taking hormonal medication that could affect their menstrual cycle, and were not shift workers.

### 2.2. Experimental protocol

Applicants were briefed about the experiment, and oral and written informed consent were obtained before the experiment. Participants completed questionnaires regarding basic information, such as age and height, and received instructions on the use of the experimental apparatus and procedures.

The participants wore a Fitbit Inspire 2 (Google, Mountain View, CA) wristwatch-type wearable device for 24 h during one menstrual cycle, except during bathing, under free-living conditions. Their TDEE, bedtime, and wake-up time were measured. Ovulation day prediction tests using ClearBlue (OMRON, Tokyo, Japan) were started 17 days before the expected start of menstruation to obtain reference data. The participants completed an electronic questionnaire every night before bedtime, reporting menstrual bleeding, ovulation-day predictor test results, and MRS scores. As the data were obtained under free-living conditions, there were no interventions regarding bedtime, wake-up time, caffeine or alcohol consumptioan, or exercise restrictions.

All study procedures were conducted in accordance with the principles of the Declaration of Helsinki. This study was approved by the Ethical Review Committee of Ritsumeikan University (approval number: BKC-LSMH-2021-063).

### 2.3. Survey items and data collection

#### 2.3.1. Basic information

Age, height, weight, and menstrual start/end dates were obtained from participants' self-reports, and body mass index was calculated based on height and weight. The MCP for each participant was determined based on these data: the menstrual phase was defined as the period of bleeding (excluding irregular bleeding) in the first half of the menstrual cycle; the follicular phase was defined as the period from the day after the last day of the menstrual phase to the day of ovulation day predictive test positivity; and the luteal phase was defined as the period from the day after ovulation day predictive test positivity to the day before the next menstrual period start date (15). Because the length of the menstrual period varies among individuals, the self-reported menstrual period was used (16). The reference data for the day of ovulation were the days after the first positive result in the ovulation day prediction test. Home luteinizing hormone tests are widely used to detect ovulation and determine fertility (17).

#### 2.3.2. Physical activity parameter: total daily energy expenditure

TDEE as a physical activity parameter was measured by Fitbit Inspire 2. The device is suitable for measuring TDEE (18). This device combines basal metabolic rate, activity data, and heart rate data to estimate TDEE (19). Data were obtained using the Fitbit Web API.

#### 2.3.3. Sleep timing parameter: midpoint of sleep time

MS time as a sleep timing parameter was measured by Fitbit Inspire 2. MS time was calculated by identifying the time halfway between bedtime and waketime. For example, the MS time is 4:00 am if sleep onset is at 00:00, and the wake-up time is 8:00 am (20). MS time has been shown to be positively correlated with the circadian rhythm phase and is often used as a surrogate for the circadian rhythm phase (21). This device estimates sleep stages by analyzing patterns of movement and heart rate, determining sleep when there is no movement detected for approximately one hour (22). Prior research on this device has indicated it to have high sensitivity (93.9%) and accuracy (76%), and its use as an objective tool for measuring sleep in daily life is considered appropriate (23).

#### 2.3.4. MRS parameters: MRS questionnaire score

The modified menstrual distress questionnaire (mMDQ), a 35-item Japanese version of the questionnaire, was used to collect MRS data. The questionnaire consists of six subscales: pain (six items), concentration symptoms (eight items), behavioral change symptoms (five items), autonomic reaction symptoms (four items), water retention (four items), and negative affect (eight items). Responses were scored on a 6-point Likert scale ranging from 1 (no response at all) to 6 (acute or partially disabling), with higher total scores indicating more severe symptoms. The original version of the MDQ was developed to assess MRS scores during menstruation, one week before menstruation, and the rest of the menstrual cycle (4, 24). In Japan, the two domains of arousal and control are often examined in six parts because there are few complaints in these domains (25). Therefore, 35 items from six environments were used in this study. In general, retrospective methods have limited reliability because the incidence and severity of symptoms are substantial and are often related to social conventions and attitudes (26). Therefore, we employed a direct method in which respondents answered the day's symptoms every day upon entering bed. The total score on each subscale of the mMDQ was used as a parameter for the MRSs.

### 2.4. Statistical analysis

The following two criteria were set for the data to be analyzed: a daily rate of at least 80% for obtaining bedtime/wake-up time information via Fitbit and a response rate of at least 90% for the electronic survey at bedtime. These criteria were used to ensure the reliability and accuracy of the data.

All statistical analyses were performed using R (R Foundation, Vienna, Austria). The significance level was set at *p* < 0.05. The generalized linear mixed model (GLMM) examined the relationship between MRSs and TDEE/MS time according to the MCP during one menstrual cycle. The objective variables for each model are as follows: Model 1, pain; Model 2, concentration symptoms; Model 3, behavioral change symptoms; Model 4, autonomic reaction symptoms; Model 5, water retention; Model 6, negative affect. The fixed effects were the same for all models and are as follows: main effects: TDEE, MS time, menstrual and luteal phases; reciprocal action: TDEE*Menstrual phase, TDEE*Luteal phase, MS time*Menstrual phase, MS time*Luteal phase. To avoid multicollinearity, the follicular phase was used as the criterion. Because of the significant individual differences in these effects, we employed a GLMM, which can be estimated more appropriately based on the random effects of human variation (27). To evaluate whether MCP acts causally, we established an interaction effect. Table 1 lists the targets and main effects of each GLMM. In this study, the Bonferroni correction was applied to adjusted *P*-values to prevent inflation of type I errors.

**Table 1:** Target and main effects for each GLMM.

Model no.	Target	Fixed factors
Model 1	Total pain score	TDEE, MS time, Menstrual phase, Luteal phase, TDEE*Menstrual phase, TDEE*Luteal phase, MS time*Menstrual phase, MS time*Luteal phase
Model 2	Total concentration score	
Model 3	Total behavioral change score	
Model 4	Total autonomic reactions score	
Model 5	Total water retention score	
Model 6	Total negative affect score	

GLMM, generalized linear mixed model; TDEE, total daily energy expenditure; MS time, midpoint of sleep time.

## 3. Results

### 3.1. Characteristics of the analysis subjects

Nine of the 14 women were included in the analysis, two of whom may have been anovulatory based on the results of ovulation day predictor tests. Three other women were excluded from the analysis because their Fitbit bedtime/waketime data acquisition rate was less than 80%. The average and standard deviations of the menstrual cycles of the analyzed subjects were 30.6 ± 3.7 days, all within the normal range. They were not pregnant. Table 2 summarizes the mean TDEE, MS time, and mMDQ subscale scores for each MCP in all participants. The mean TDEE was 1,757.2 ± 246.9 kcal for the menstrual phase, 1,717.0 ± 221.9 kcal for the luteal phase, and 1,698.9 ± 215.5 kcal for the follicular phase. The mean MS time was 5.49 ± 1.31 h for the luteal phase, 5.35 ± 1.70 h for the menstrual phase, and 5.29 ± 1.52 h for the follicular phase. The menstrual phase group had the highest, and the follicular phase group had the lowest total mMDQ scores on all subscales.

**Table 2:** Average and standard deviation of TDEE, MS time, and mMDQ scores per MCP for all participants.

MCP		Menstrual phase	Follicular phase	Luteal phase
TDEE [kcal]		1,757.2 ± 246.9	1,698.9 ± 215.5	1,717.0 ± 221.9
MS time [h]		5.35 ± 1.70	5.29 ± 1.52	5.49 ± 1.31
The score of the mMDQ [point]	Pain	10.2 ± 3.0	8.2 ± 2.0	8.5 ± 2.3
	Concentration	12.1 ± 5.4	10.7 ± 3.8	11.1 ± 4.8
	Behavioral change	8.8 ± 3.8	8.3 ± 3.7	8.6 ± 3.2
	Autonomic reactions	5.4 ± 1.4	4.5 ± 0.7	4.8 ± 0.9
	Water retention	6.5 ± 2.0	5.4 ± 1.4	6.4 ± 1.7
	Negative affect	11.8 ± 4.5	8.8 ± 1.1	10.2 ± 3.5

TDEE, total daily energy expenditure; MS time, midpoint of sleep time; mMDQ, modified menstrual distress questionnaire; MCP, menstrual cycle phase.

### 3.2. Relationship evaluation using GLMM

The following were the results of the relationship between MRSs and TDEE/MS time according to the MCP using GLMM. The probability distribution was gamma, and the link function was logarithmic. Tables 3, 4 show the results for the GLMM fixed coefficients.

**Table 3:** Fixed coefficients of models 1–4.

Target	Fixed factors	Estimate	SE	*t* value	Adjusted *p*-value
Model 1 Pain	Intercept	1.9**	0.54	3.6	0.0024
	TDEE	0.000080	0.000130	0.62	1.0
	MS time	−0.0069	0.042	−0.16	1.0
	Menstrual phase	0.75***	0.19	3.9	<0.001
	Luteal phase	0.49**	0.14	3.5	0.0036
	TDEE*Menstrual phase	−0.000017	0.000058	−0.29	1.0
	TDEE*Luteal phase	−0.00011	0.000042	−2.6	0.081
	MS time*Menstrual phase	−0.10*	0.029	−3.5	0.0047
	MS time*Luteal phase	−0.049	0.020	−2.5	0.11
Model 2 Concentration	Intercept	1.9***	0.22	8.4	<0.001
	TDEE	0.00016	0.00007	2.3	0.20
	MS time	0.032	0.023	1.4	1.0
	Menstrual phase	1.1***	0.14	7.8	<0.001
	Luteal phase	0.84***	0.11	7.9	<0.001
	TDEE*Menstrual phase	−0.00035***	0.000043	−8.2	<0.001
	TDEE*Luteal phase	−0.00039***	0.000032	−12	<0.001
	MS time*Menstrual phase	−0.076***	0.019	−3.9	<0.001
	MS time*Luteal phase	−0.035	0.016	−2.2	0.24
Model 3 Behavioral change	Intercept	2.9***	0.31	9.5	<0.001
	TDEE	−0.00061***	0.00013	−4.5	<0.001
	MS time	0.024	0.035	0.67	1.0
	Menstrual phase	−0.42	0.21	−2.0	0.43
	Luteal phase	0.12	0.16	0.76	1.0
	TDEE*Menstrual phase	0.00041***	0.000072	5.7	<0.001
	TDEE*Luteal phase	0.000030	0.000051	0.60	1.0
	MS time*Menstrual phase	−0.038	0.026	−1.4	1.0
	MS time*Luteal phase	−0.023	0.021	−1.1	1.0
Model 4 Autonomic reactions	Intercept	1.40***	0.26	5.4	<0.001
	TDEE	0.000027	0.000076	0.36	1.0
	MS time	0.008	0.074	0.11	1.0
	Menstrual phase	0.72***	0.16	4.4	<0.001
	Luteal phase	0.69***	0.12	5.8	<0.001
	TDEE*Menstrual phase	−0.00019**	0.000052	−3.7	0.0021
	TDEE*Luteal phase	−0.00032***	0.000037	−8.7	<0.001
	MS time*Menstrual phase	−0.053	0.022	−2.4	0.15
	MS time*Luteal phase	−0.019	0.017	−1.1	1.0

SE, standard error; TDEE, total daily energy expenditure; MS time, midpoint of sleep time.

* Significant at the *p* < 0.05 level.

** Significant at the *p* < 0.01 level.

*** Significant at the *p* < 0.001 level.

**Table 4:** Fixed coefficients of models 5–6.

Target	Fixed factors	Estimate	SE	*t* value	Adjusted *p*-value
Model 5 Water retention	Intercept	1.4***	0.23	6.1	<0.001
	TDEE	0.000077	0.000094	0.82	1.0
	MS time	0.026	0.064	0.40	1.0
	Menstrual phase	0.68**	0.18	3.7	0.0023
	Luteal phase	0.94***	0.14	6.8	<0.001
	TDEE*Menstrual phase	−0.00011	0.000063	−1.7	0.78
	TDEE*Luteal phase	−0.00033***	0.000045	−7.3	<0.001
	MS time*Menstrual phase	−0.067*	0.023	−2.9	0.036
	MS time*Luteal phase	−0.042	0.019	−2.2	0.27
Model 6 Negative affect	Intercept	1.9***	0.18	10	<0.001
	TDEE	0.00013	0.000060	2.2	0.23
	MS time	0.019	0.021	0.93	1.0
	Menstrual phase	1.4***	0.20	7.1	<0.001
	Luteal phase	1.0***	0.15	6.8	<0.001
	TDEE*Menstrual phase	−0.00038***	0.000066	−5.7	<0.001
	TDEE*Luteal phase	−0.00033***	0.000046	−7.1	<0.001
	MS time*Menstrual phase	−0.10**	0.026	−3.7	0.0016
	MS time*Luteal phase	−0.064*	0.021	−3.1	0.017

SE, standard error; TDEE, total daily energy expenditure; MS time, midpoint of sleep time.

* Significant at the *p* < 0.05 level.

** Significant at the *p* < 0.01 level.

*** Significant at the *p* < 0.001 level.

#### 3.2.1. Relationship between pain and TDEE/MS time according to the MCP

**Figure 1:**
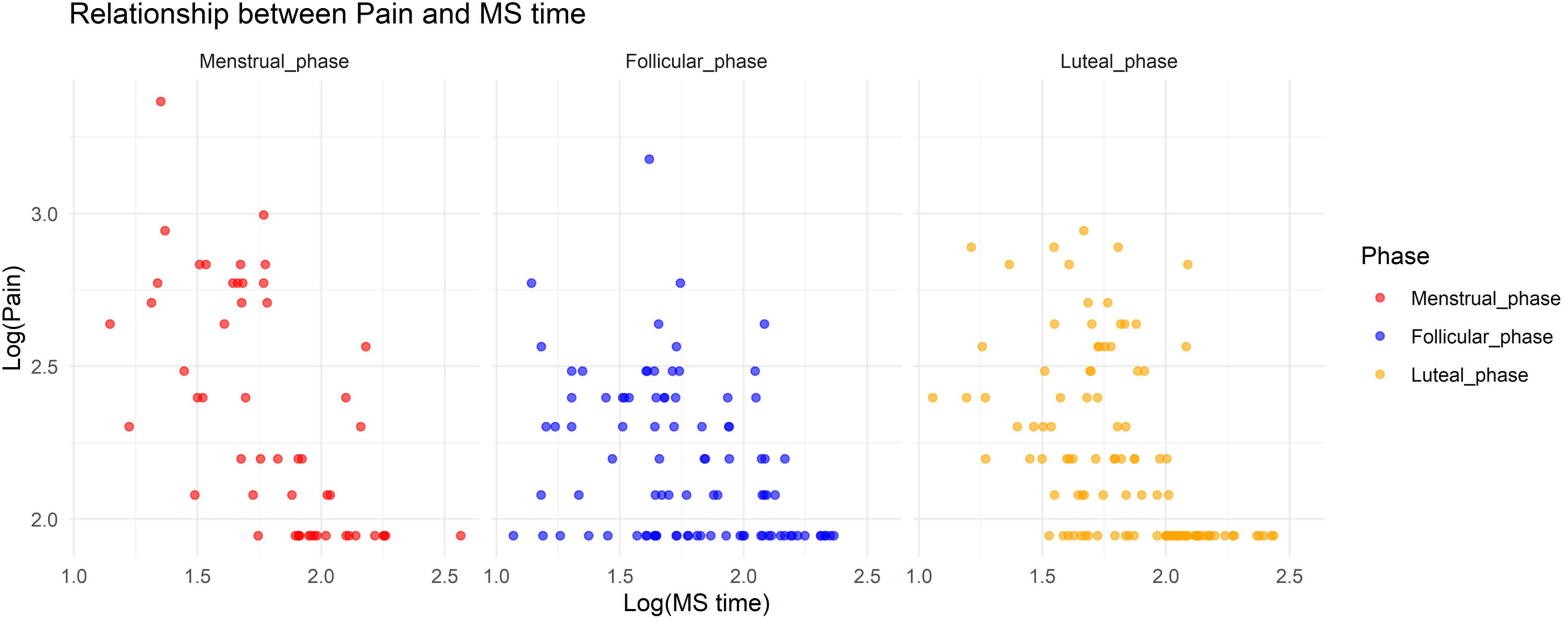
Relationship between pain and midpoint of sleep time.

In Model 1, where the target variable was pain, we observed a significant increase in pain during the menstrual and luteal phases compared to the follicular phase (Menstrual phase: Estimate = 0.75, standard error [SE] = 0.19, adjusted *p* < 0.001; Luteal phase: Estimate = 0.49, SE = 0.14, adjusted *p* = 0.0036). Additionally, there was a notable interaction effect between MS time and the menstrual phase, with a decrease in pain associated with a delayed MS time during the menstrual phase (Estimate = −0.10, SE = 0.029, adjusted *p* = 0.0047). Figure 1 shows the relationship between pain and MS time.

#### 3.2.2. Relationship between concentration symptoms and TDEE/MS time according to the MCP

**Figure 2:**
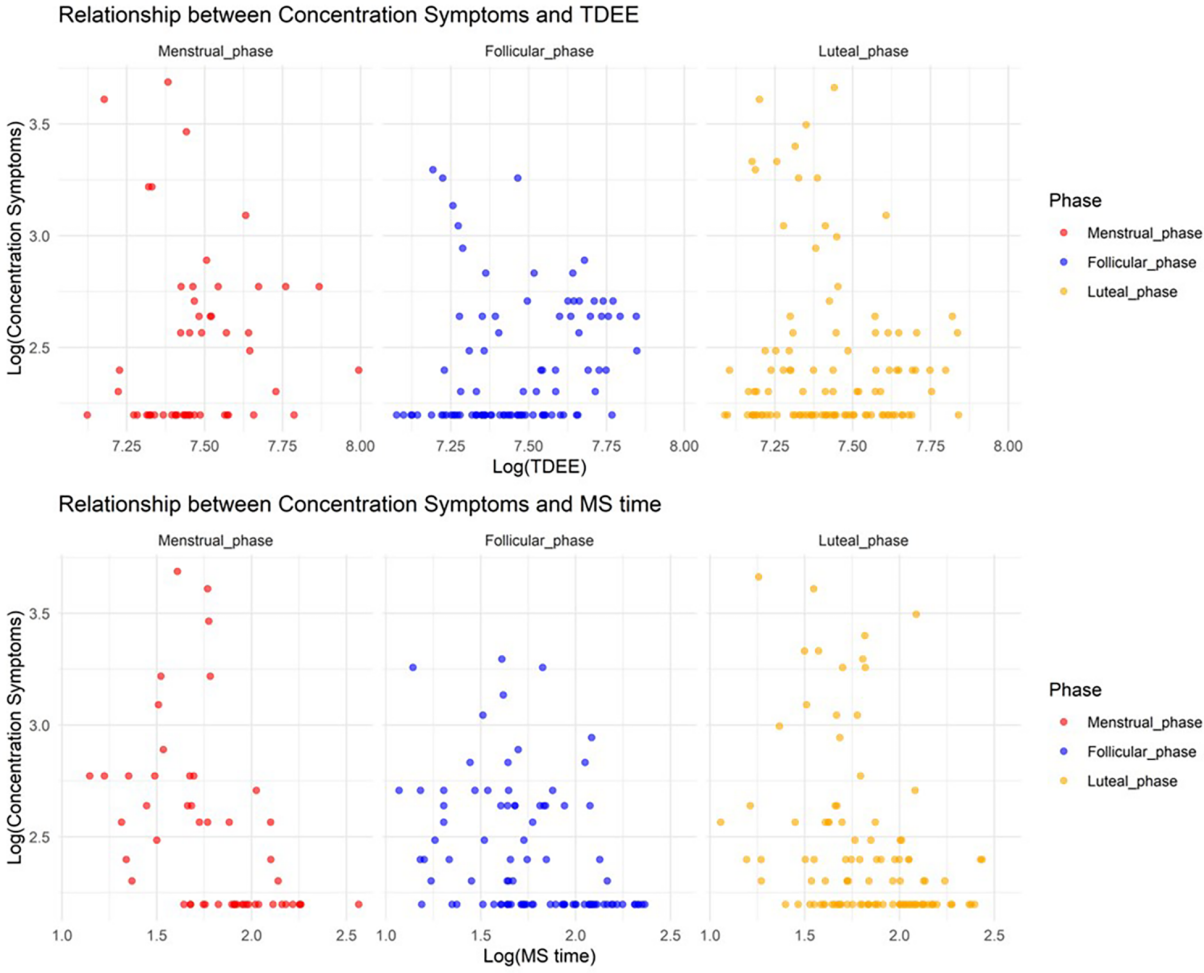
Relationship between concentration symptoms and total daily energy expenditure/midpoint of sleep time.

In Model 2, where the target variable was concentration symptoms, we observed a significant increase in concentration symptoms during the menstrual and luteal phases compared to the follicular phase (Menstrual phase: Estimate = 1.1, SE = 0.14, adjusted *p* < 0.001; Luteal phase: Estimate = 0.84, SE = 0.11, adjusted *p* < 0.001). Furthermore, an increase in TDEE during these phases was associated with a decrease in concentration symptoms (TDEE* Menstrual phase: Estimate = –0.00035, SE = 0.000043, adjusted *p* < 0.001; TDEE*Luteal phase: Estimate = –0.00039, SE = 0.000032, adjusted *p* < 0.001). Additionally, a regression in MS time during the menstrual phase was related to a reduction in concentration symptoms (MS time* Menstrual phase: Estimate = –0.076, SE = 0.019, adjusted *p* < 0.001). Figure 2 shows the relationship between concentration symptoms and TDEE/MS time.

#### 3.2.3. Relationship between behavioral change symptoms and TDEE/MS time according to the MCP

**Figure 3:**
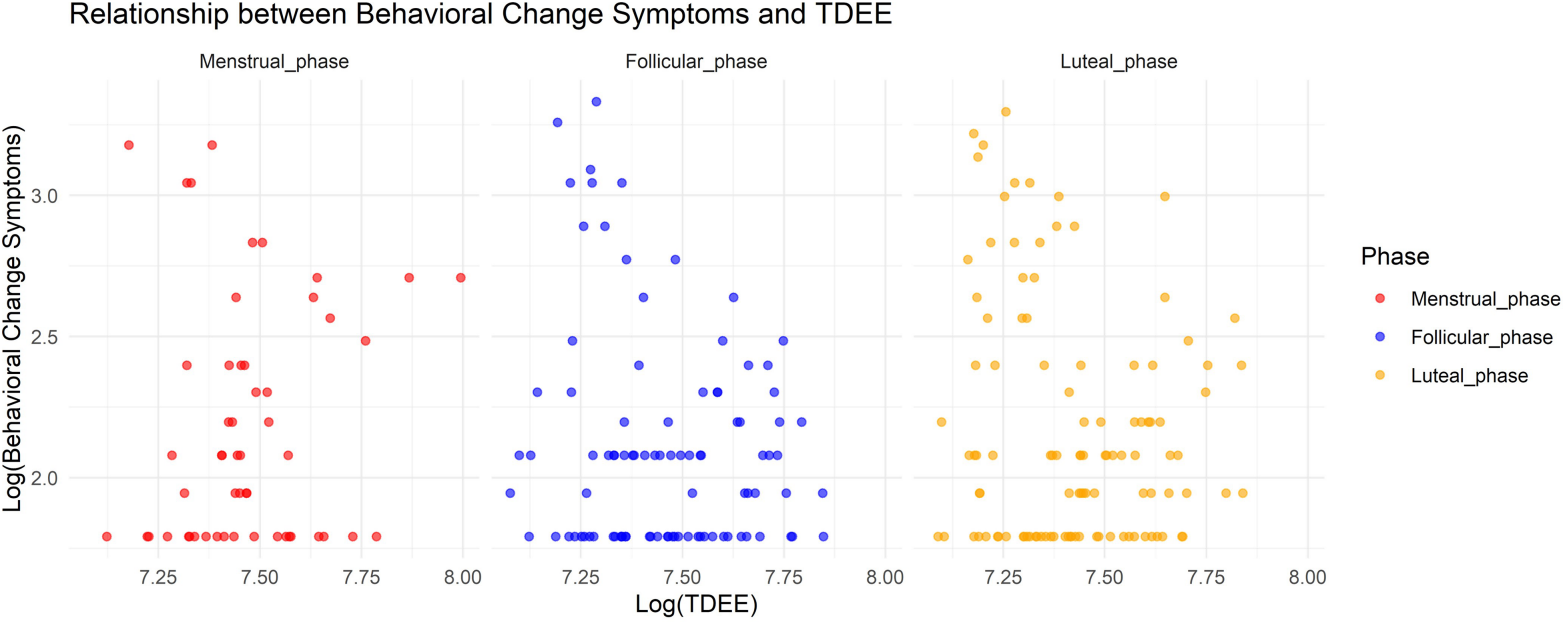
Relationship between behavioral change symptoms and total daily energy expenditure.

In Model 3, where the target variable was behavioral change symptoms, we observed a significant association with TDEE, where an increase in TDEE was linked with a decrease in behavioral change symptoms (Estimate = –0.00061, SE = 0.00013, adjusted *p* < 0.001). Moreover, the interaction term between TDEE and the menstrual phase was significant, indicating that an increase in TDEE during the menstrual phase is associated with an increase in behavioral change symptoms (Estimate = 0.00041, SE = 0.000072, adjusted *p* < 0.001). No significant effects were found for the menstrual and luteal phases on their own, nor for their interactions with TDEE. Figure 3 shows the relationship between behavioral change symptoms and TDEE.

#### 3.2.4. Relationship between autonomic reaction symptoms and TDEE/MS time according to the MCP

**Figure 4:**
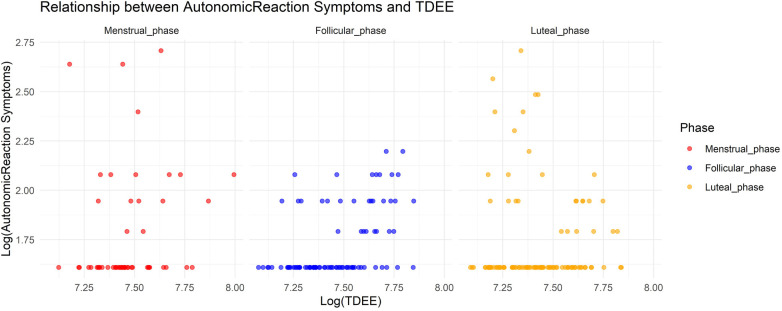
Relationship between autonomic reaction symptoms and total daily energy expenditure.

In Model 4, where the target variable was autonomic reaction symptoms, we observed a significant increase in autonomic reaction symptoms during the menstrual and luteal phases compared to the follicular phase (Menstrual phase: Estimate = 0.72, SE = 0.16, adjusted *p* < 0.001; Luteal phase: Estimate = 0.69, SE = 0.12, adjusted *p* < 0.001). Furthermore, an increase in TDEE during these phases was associated with a decrease in autonomic reaction symptoms (TDEE* Menstrual phase: Estimate = –0.00019, SE = 0.000052, adjusted *p* = 0.0021; TDEE*Luteal phase: Estimate = –0.00032, SE = 0.000037, adjusted *p* < 0.001). Figure 4 shows the relationship between autonomic reaction symptoms and TDEE.

#### 3.2.5. Relationship between water retention and TDEE/MS time according to the MCP

**Figure 5:**
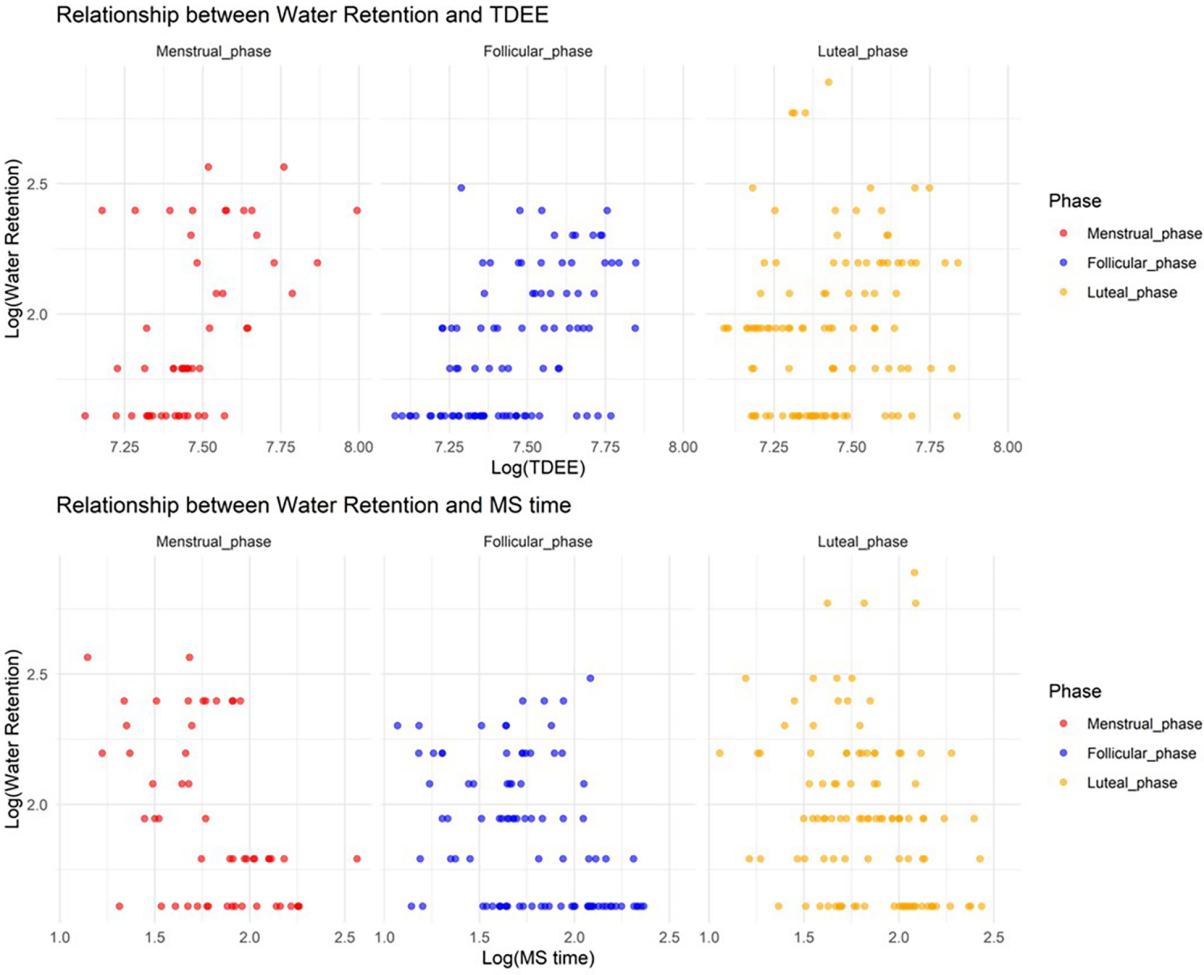
Relationship between water retention and total daily energy expenditure/midpoint of sleep time.

In Model 5, where the target variable was water retention, we observed a significant increase in water retention during the menstrual and luteal phases compared to the follicular phase (Menstrual phase: Estimate = 0.68, SE = 0.18, adjusted *p* = 0.0023; Luteal phase: Estimate = 0.94, SE = 0.14, adjusted *p* < 0.001). Furthermore, an increase in TDEE during the luteal phase was associated with a decrease in water retention (TDEE*Luteal phase: Estimate = –0.00033, SE = 0.000045, adjusted *p* < 0.001). Additionally, a regression in MS time during the menstrual phase was related to a reduction in water retention (MS time* Menstrual phase: Estimate = –0.067, SE = 0.023, adjusted *p* = 0.036). Figure 5 shows the relationship between water retention and TDEE/MS time.

#### 3.2.6. Relationship between negative affect and TDEE/MS time according to the MCP

**Figure 6:**
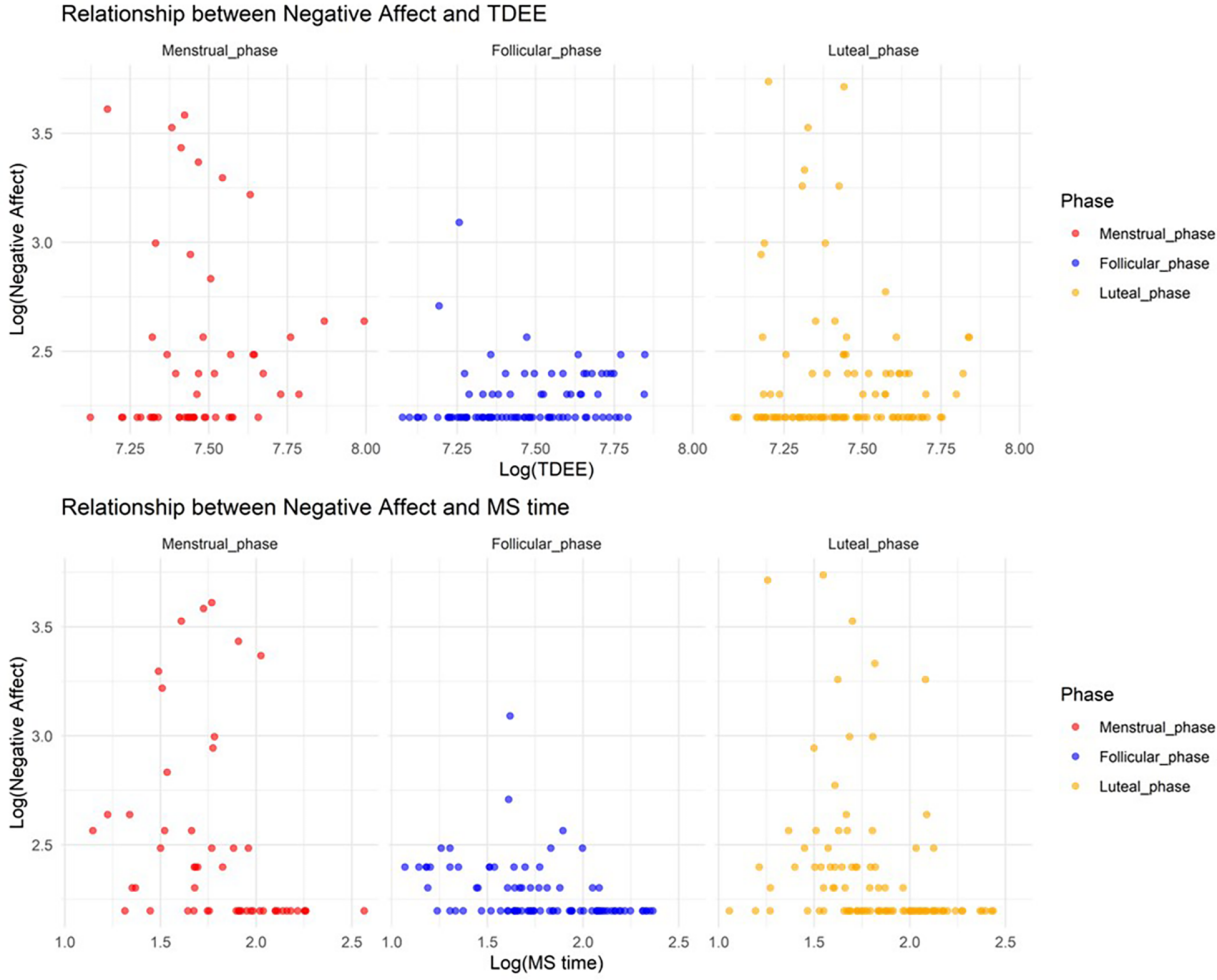
Relationship between negative affect and total daily energy expenditure/midpoint of sleep time.

In Model 6, where the target variable was negative affect, we observed a significant increase in negative affect during the menstrual and luteal phases compared to the follicular phase (Menstrual phase: Estimate = 1.4, SE = 0.20, adjusted *p* < 0.001; Luteal phase: Estimate = 1.0, SE = 0.15, adjusted *p* < 0.001). Furthermore, an increase in TDEE during these phases was associated with a decrease in negative affect (TDEE* Menstrual phase: Estimate = –0.00038, SE = 0.000066, adjusted *p* < 0.001; TDEE*Luteal phase: Estimate = –0.00033, SE = 0.000046, adjusted *p* < 0.001). Additionally, a regression in MS time during these phases was related to a reduction in negative affect (MS time* Menstrual phase: Estimate = –0.10, SE = 0.026, adjusted *p* = 0.0016; MS time*Luteal phase: Estimate = –0.064, SE = 0.021, adjusted *p* = 0.017). Figure 6 shows the relationship between negative affect and TDEE/MS time.

### 3.3. Summary of results

This study evaluated the relationship between MRSs (pain, concentration symptoms, behavioral change symptoms, autonomic reaction symptoms, water retention, and negative affect) and TDEE, as well as MS time according to the MCP. Firstly, all MRSs, except behavioral change symptoms, were significantly more pronounced during the menstrual and luteal phases compared to the follicular phase. Secondly, delayed MS time was associated with reduced pain, concentration symptoms, water retention, and negative affect during the menstrual phase, and reduced negative affect during the luteal phase. Finally, an increase in TDEE was associated with reduced concentration symptoms, autonomic reaction symptoms, and negative affect during the menstrual and luteal phases and with reduced water retention only during the luteal phase.

## 4. Discussion

This study investigated the relationship between MRSs and TDEE as well as MS time according to the MCP. The findings support the hypothesis that MRS severity is associated with variation in TDEE and MS time according to the MCP.

Consistent with hormonal fluctuation theories, our results indicate that MRSs (except behavioral change symptoms) are significantly more severe during the menstrual and luteal phases. The elevated presence of MRSs (pain, concentration symptoms, autonomic reaction symptoms, water retention, and negative affect) during these phases could be attributed to the hormonal changes in the fluctuations in estrogen and progesterone levels. The results of the present study are consistent with those of previous studies showing increased scores of mMDQ subscales in the menstrual phase group compared with those in the follicular phase group. Previous studies have shown that women generally experience unpleasant MRSs, and the results of the present study confirmed this (24). Furthermore, in a survey of 200 female college students, many reported premenstrual syndrome appearing during the luteal phase, although the degree of severity varied (28). The lack of significant variance in behavioral change symptoms across the MCP may suggest the predominance of external factors such as daily stressors over hormonal influences, a hypothesis that warrants further investigation.

A noteworthy finding is that delayed MS time was associated with reduced pain, concentration symptoms, water retention, and negative affect during the menstrual phase, and reduced negative affect during the luteal phase. This suggests that sleep timing may play a critical role in the exacerbation or alleviation of MRSs. Recent reports have indicated that women with dysmenorrhea experience higher levels of oxidative stress than healthy individuals (29). Melatonin has antioxidant and anti-inflammatory effects (30). Oral administration of melatonin at bedtime during menstruation has been suggested to alleviate the pain associated with dysmenorrhea and improve sleep quality (31, 32). Water retention during the menstrual phase is thought to be due to changes in the estrogen and progesterone levels. The delayed MS time may have positively affected sleep quality and reduced concentration symptoms and water retention. Therefore, regression of the melatonin secretion rhythm and prolonging the secretion time due to late awakening may be involved in alleviating the symptoms of pain and water retention during menstruation. A previous study reported that women with PMDD tended to have advanced circadian rhythm acrophase (13). Our study showed that there was a relationship between decreased negative affect and delayed MS time in both the luteal and menstrual phases. On the other hand, previous studies have indicated an association between the magnitude of social jet lag, circadian rhythm disturbances, and MRSs (14). Therefore, it is considered important to maintain a daily rhythm according to the MCP, to delay MS time during periods when MRSs are severe, and to advance the MS time when they are not. There was no relationship between autonomic reactions and MS time during the menstrual phase because this symptom has large temporal variability and requires more precise assessment. More objective methods for assessing this relationship, such as electroencephalography, would provide a more accurate understanding.

Our study revealed that an increase in TDEE was associated with reduced concentration symptoms, autonomic reaction symptoms, and negative affect during the menstrual and luteal phases and reduced water retention during the only luteal phase. This was considered to be because exercise stimulates estradiol secretion in the body and serotonin secretion in the brain (11, 12). On the other hand, it may be the result of sex hormones affecting TDEE. In an online survey, approximately 40% of respondents reported reduced activity levels during the menstrual phase (33). In the luteal phase, a significant increase in average core body temperature and energy expenditure were observed (34).

In this study, physical activity and sleep timing were monitored using a wearable device during one menstrual cycle, allowing for more objective data to be collected. In a previous study, physical activity measured using wearable devices was found to be more reliable than self-reported physical activity (35). Furthermore, we provided integrated models that consider the relationship between MRSs and physical activity/sleep timing according to the MCP during daily life, allowing the relationship to be evaluated under real-life conditions.

This study has some limitations. Firstly, the relatively small sample size of our study may limit the robustness and generalizability of our findings. A larger and more diverse cohort would enhance the statistical power and enable more definitive conclusions about the complex interactions we have explored. Secondly, our analysis did not include chronotype measures. Among university students, the “eveningness” chronotype is associated with MRSs (36). The absence of this variable may have restricted our understanding of the nuances in the relationship between MRSs and sleep timing. Another limitation lies in the scope of our study, which was conducted over a single menstrual cycle. The cyclical nature of menstrual symptoms and their modulators may be better understood through longitudinal studies spanning multiple cycles, providing a richer, more detailed analysis of these patterns over time. Future research should aim to include a more diverse demographic and extend its scope to encompass other influential lifestyle factors such as diet, stress, and hormonal fluctuations, which could play a significant role in MRSs.

In conclusion, this study clarified the relationship between MRSs and physical activity/sleep timing according to the MCP. This study evaluated the relationship between MRSs and TDEE as well as MS time according to the MCP. Delayed MS time was associated with reduced pain, concentration symptoms, water retention, and negative affect during the menstrual phase and reduced negative affect during the luteal phase. Increased TDEE was associated with reduced concentration symptoms, autonomic reaction symptoms, and negative affect during the menstrual and luteal phases and reduced water retention only during the luteal phase. In Japan, 74.0% of women experience MRSs, and the annual socioeconomic loss due to MRSs is estimated to be approximately 682.8 billion yen (4). Future research must be done to clarify the guidelines for proper physical activity and sleep timing according to the MCP, thereby improving women's health management. Furthermore, the possibility of a tailor-made health management approach utilizing wearable devices has been suggested. The findings of this study can be used to develop applications, services, and workstyles in the future. Many countries have begun to adopt teleworking and staggered working hours after coronavirus disease 2019 (37). One specific application is the provision of applications that support the optimization of sleep and activity according to an individual's MCP. Combining this with wearable devices that estimate ovulation periods would also be adequate (38). This would enable women to better understand their physical condition, physical activity, and sleep timing and take appropriate non-therapy measures to alleviate MRSs.

## Data Availability

The datasets presented in this article are not readily available because the ethical review of our study permits the use of the data only for this research and does not permit the release of the data. If you wish to obtain data as in this study, please contact us through collaborative research. Requests to access the datasets should be directed to HM, rr0095ie@ed.ritsumei.ac.jp.
